# Parenting Practices as Risk or Preventive Factors for Adolescent Involvement in Cyberbullying: Contribution of Children and Parent Gender

**DOI:** 10.3390/ijerph15122664

**Published:** 2018-11-27

**Authors:** Olga Gómez-Ortiz, Eva M. Romera, Rosario Ortega-Ruiz, Rosario Del Rey

**Affiliations:** 1 Department of Psychology, Universidad de Córdoba, 14071 Córdoba, Spain; eva.romera@uco.es (E.M.R.); ortegaruiz@uco.es (R.O.-R.); 2 Department of Educational and Developmental Psychology, Universidad de Sevilla, 41013 Sevilla, Spain; delrey@us.es

**Keywords:** parental discipline, family, cyber-victimization, social networks, gender differences

## Abstract

Literature points out the role of parenting on adolescent cyberbullying involvement. However, it is necessary to clarify how gender affects this relationship. The aim of this study has been to examine the relation between the adolescents’ perception about parenting practices, and their involvement in cyberbullying, bearing in mind both girls’ and boys’ gender and progenitors’ gender. The sample comprised 2060 Spanish secondary school students (47.9% girls; Mage = 14.34). Two-way ANOVA and binary logistic regression analyses were carried out. An effect of the interaction between sex and cyberbullying roles in maternal affection and communication, inductive discipline, and psychological control, as well as paternal promotion of autonomy and psychological control, was found. In general, it can be observed that the more negative results were found in cyber-aggressors, especially when this role is assumed by girls. The results of logistic regression analysis suggest that parenting practices explain better cyberbullying involvement in girls compared to boys, finding some important differences between both sexes regarding protective and risk factors. These findings highlight the importance of parenting practices to explain cyberbullying involvement, which supports the necessity of including family among the addresses of intervention programs.

## 1. Introduction

In recent years, we have witnessed a revolution in the development of information and communication technologies (ICT) that has entailed drastic changes, affecting our behaviour and our way of communicating. This transformation, which has especially affected the new generations [1], has provided us with a high number of resources that make our life easier and offer us development potentialities, but which can also harm ourselves and others [2]. In this regard, and in parallel with the technological evolution, the number of studies on cyberbullying has increased, understanding it as a way of interpersonal violence produced through the ICT use among children and adolescents [3].

Smith et al. [4] describe cyberbullying as “an aggressive intentional act that is carried out by a group or an individual repeatedly and over time against a victim who cannot easily defend him or herself” (p. 376). This definition emphasises the repetition, the aggressor’s intention and the power imbalance between him/her and the victim, as those elements that define cyberbullying, following the characteristics that Olweus [5] and other authors [6,7] already outlined of the phenomenon of bullying. In addition, cyberbullying presents characteristics that are slightly different from traditional bullying, such as having a great and asynchronous audience and the aggressor’s major possibility of being anonymous [8,9]. Due to these characteristics, the damage suffered by a cyber-victim could be even greater than the one suffered from a victim of traditional bullying [3,10,11]. 

Intrapersonal and interpersonal factors have been shown to be essential in the prevention of aggression or victimization through ICT [12]. Certain contextual aspects, such as the school environment, may also condition the development of this phenomenon [13]. Yet, the influence of the family context has not been explored in depth, as most research has focused on analysing the parental norms or the progenitors’ control and supervision regarding the use of ICT at home, which has been named as “parental mediation” [14]. The parental mediation has proved to be useful when preventing the addiction to internet and the involvement in cyberbullying, especially cyber-victimization [15,16,17], although its efficacy is limited unless it meets adequate and positive educational standards [18].

Research about parenting and cyberbullying involvement have been developed according to two different perspectives. The first has focused on general parenting styles defined in terms of warmth and demandingness [19,20]. According to this perspective, the democratic and the indulgent parenting styles—both characterized by the use of warmth practices and reasoning, although the first use the control more than the second—are those which seem to prevent the involvement in cyberbullying better. On the contrary, the authoritarian style—characterized by the use of coercion and privation practices—is positively related to the possibility of becoming an aggressor or victim of this violent phenomena, especially when parents accompanied this style with the use of physical discipline and high control [21,22,23,24]. The second perspective examined more than general parenting styles, specific parental practices which can be part of previous parenting styles, such as affection, communication, discipline or control, and others which, traditionally, have not been included in the configuration of parenting styles [25]. Between these last practices are the promotion of autonomy and humour [26]. Furthermore, some specific children behaviours are included in this last perspective because they are related to parenting, such as children disclosure, which let parents control their children indirectly [27,28,29]. The available studies on the analysis of parental practices regarding involvement in cyberbullying point at the low parental affection or support as an important risk factor, especially for cyber-victims [18,22,30,31,32]. The cyber-aggressors’ progenitors are usually described as barely committed to the supervision and establishment of limits and highly prone to use punitive or severe disciplinary procedures [23,24,33]. Moreover, a certain level of parental intrusiveness has been linked to the development of aggressive behaviours. Therefore, information on the children’s and adolescents’ online behaviour—obtained by requesting their progenitors through direct questions—has been associated with higher levels of cyber-aggression [34]. The effects of control on cyber-victimization are not so conclusive and they do not seem to act like cyber-aggression, registering studies that describe control practices as a mild, but significant, risk factor [35]. Family communication has been related to a lower involvement in cyberbullying, above all, when +this communication entails a voluntary disclosure by the minor, informing his/her parents about the activities s/he does on the internet [36,37]. A recent study has shown the connection between involvement in cyberbullying and the parental promotion of autonomy, and psychological control. The parental promotion of autonomy is indirectly related to this phenomenon through the development of empathy and feelings of human connection. Nevertheless, the psychological control has been described as a risk factor which increases the possibility of children and adolescents being cyber-victimised or that they attack their peers through ICT [38].

Apart from the examined parenting practices, other parental behaviours, like those related to humour and the management of discipline, have shown to be important elements explaining bullying involvement. In this sense, it has been shown that while a positive humour and the use of inductive discipline are protective factors which seem to prevent the involvement in bullying, a bad humour and the use of punitive discipline have a very negative effect for children, increasing their likelihood to be involved in this violent phenomenon [39,40]. Nevertheless, studies examining the relation of these parental practices with involvement in cyberbullying are scarce or non-existent.

Research analysing the relationship between bullying involvement and the rest of parenting practices are in line with those focused on cyberbullying, showing that the lack of affection and a low behavioural control are risk factors for the involvement in bullying, and especially for aggression. Regarding peer victimization, the most important risk factor seems to be the use of psychological control and to avoid promoting autonomy [39,41,42]. Some of these studies have shown that the influence of those parental practices on bullying involvement is different depending on teenager’s gender, being involvement of girls more conditioned by parenting practices than that of boys [39,40]. However, the research focused on cyberbullying has not examined the moderator impact of gender in an in-depth manner, being necessary to study possible differences between boys and girls.

The reviewed literature demonstrates the importance of further studying the effect of parenting practices on youngsters’ involvement in cyberbullying to clarify the effect of the understudied parental behaviours and attitudes, such as psychological control, promotion of autonomy, humour, and parental discipline. Moreover, taking into account that family does not influence in the same way on boys’ and girls’ psychosocial adjustment, and specifically on their bullying involvement [40,43], it is important to identify if these gender differences are also present in the study of cyberbullying. Taking this statement into account, the purpose of this study has been to explore the relation among involvement in different cyberbullying roles (cyber-victim, cyber-aggressor, cyber-bully/victim vs. non-involved), parenting practices (measured through affection and communication, behavioural control, psychological control, promotion of autonomy, humour, children’s disclosure, and the management of discipline), differentiating the gender of adolescents and progenitors. This purpose can be divided into other more specific objectives to examine this relationship from a descriptive and inferential perspective:

1. To analyse the possible differences in the perception of parenting practices following the role in cyberbullying and assess the possible influence of gender.
2. To explore the influence of parenting practices on the involvement in cyberbullying roles, paying attention to progenitors’ and minors’ gender.

Regarding these objectives, the following hypotheses were established. The first two hypotheses are relative to the first aim, while the last ones are related to the second aim.

1. We expect to find differences in parenting practices regarding the involvement in cyberbullying roles. In particular, non-involved minors will score higher in affection and communication, behavioural control, promotion of autonomy, humour, and disclosure. Cyber-victims, cyber-aggressors and cyber-bully/victim will score higher in psychological control and punitive parental discipline (physical punishment and psychological aggression) [22,30].
2. The differences in parenting practices according to the cyberbullying roles will be affected by teenager’s sex [39,40].
3. The parenting practices will be able to explain an important percentage of variance of involvement in cyberbullying roles, being affection and communication the practice which will show the stronger negative relationship with the involvement in the roles of cyberbullying roles, and coercive practices those which will be positively related to the involvement in this violent dynamic as a cyber-victim, cyber-aggressor, and cyber-bully/victim in a higher way [32,35].
4. The impact of parenting practices on cyberbullying involvement will be higher in girls [40].

## 2. Materials and Methods

### 2.1. Sample

To carry out this research, the reference population were the secondary education students of Andalusia—South of Spain—(368,838 students). To select a representative sample, we carried out a probabilistic random, stratified, cluster, one-stage sampling with proportional affixation [44]. Several strata were established based on the geographical area (Eastern Andalusia versus Western Andalusia), the type of centre (private and charter school versus public school), and the population of the municipality in which the centre was located (less than 10,000 inhabitants; between 10,001 and 100,000 inhabitants; more than 100,000 inhabitants). The level of confidence was 95.5% and the sample error was 2.5%, assuming the greatest variability (*p* = *q* = 0.5) [45]. Less than 2% of participants of the selected schools refused to participate in the study.

The final sample comprised 2060 secondary education students. Specifically, 52.1% of the students were boys, and 47.9%, girls. Their ages ranged from 12 to 19 (M = 14.34; DT = 1.34). Regarding the academic year, 28.4% of the students were completing their first year of secondary education, 28.4% the Second year, 22.1% the third year, and 21.1%, the fourth year. While 66.8% of the students belonged to public centres, 33.2% came from private and charter schools. Most students were born in Spain (95.9%).

All subjects gave their informed consent for inclusion before they participated in the study. The study was conducted in accordance with the Declaration of Helsinki, and the protocol was approved by the Ethics Committee of the University of Córdoba.

### 2.2. Instruments

To find out the parenting practices not related to discipline, we used the “Scale for the Assessment of the parenting styles of adolescents’ mothers and fathers” [25]. This instrument is made up of 82 items—41 items related to the father’s style and 41 items associated with the mother’s style—which are answered through a Likert-type scale with six response options following the degree of agreement (1 = strongly disagree; 6 = completely agree). This scale is structured in six factors: (1) affection and communication (e.g., “S/he enjoys speaking about different things with me”); (2) behavioural control (e.g., “S/he asks me on what I spend my money”); (3) psychological control (e.g., “S/he is always telling me what I have to do”); (4) promotion of autonomy (e.g., “S/he encourages me to make my own decisions”); (5) humour (e.g., “S/he usually tells me jokes”); (6) minor’s disclosure (e.g., “I tell him/her what I do in my free time”). In this research, the internal consistency (assessed with McDonald’s Omega and Cronbach’s Alpha, respectively) was adequate for each dimension: ω*_h_*1mother = 0.93 (*α* = 0.90); ω*_h_*1father = 0.94 (*α* = 0.92); ω*_h_*2mother = 0.85 (*α* = 0.80); ω*_h_*2father = 0.87 (*α* = 0.83); ω*_h_*3mother = 0.87 (*α* = 0.83); ω*_h_*3father = 0.87 (*α* = 0.83); ω*_h_*4mother = 0.89 (*α* = 0.86); ω*_h_*4father = 0.87 (*α* = 0.87); ω*_h_*5mother = 0.90 (*α* = 0.88); ω*_h_*5father = 0.91 (*α* = 0.89); ω*_h_*6mother = 0.87 (*α* = 0.82); ω*_h_*6father = 0.88 (*α* = 0.85). The internal consistency for the general scale was also adequate (ω*_h_* = 0.95; *α* = 0.93).

The assessment of the parental discipline was carried out with the inventory of discipline dimensions, in the children’s and adolescents’ version called “Discipline Dimensions Inventory” [46]. This instrument has been validated in the Spanish population by Calvete, Gámez-Guadix and Orue [47] and comprises 52 items—26 related to the father’s discipline and 26 related to the mother’s discipline—which are completed through a Likert-type scale with 10 response options to categorise the frequency which minors expect their progenitors to have done any of the proposed actions (0 = never; 9 = twice or more a day). The questionnaire is organised into four factors which, at the same time, can be divided into more specific disciplinary behaviours. The first one is called inductive discipline (ID) and includes distraction, explanation, and reward behaviours (e.g., “You have been explained what the rules were to avoid repeating a bad action”). The second one, called supervision (SUP), refers to the surveillance behaviours or those behaviours related to ignoring children’s behaviour (e.g., “You were not paid attention on purpose when you misbehaved”). The third one, called punishment (PUN), covers not only the physical but also the psychological punishment (e.g., “You were flogged, slapped or smacked”). Finally, the response cost (RC) factor comprises those behaviours that would entail a compensation for any damage or a withdrawal of privileges (e.g., “You were forced to do something to compensate the damaged caused due to a misbehaviour, for example, paying for a broken glass”). In this research, the assessment of internal consistency was adequate not only for the general scale (ω*_h_* = 0.95; *α* = 0.96), but also for each of the dimensions: ω*_h_*ID-mother = 0.75 (*α* = 0.73); ω*_h_*ID-father = 0.78 (*α* = 0.74); ω*_h_*SUP-mother = 0.76 (*α* = 0.74); ω*_h_*SUP-father = 0.75 (*α* = 0.69); ω*_h_*PUN-mother = 0.92 (*α* = 0.87); ω*_h_*PUN-father = 0.92 (*α* = 0.88); ω*_h_*RC-mother = 0.86 (*α* = 0.82); ω*_h_*RC-father = 0.87 (*α* = 0.82).

Involvement in cyberbullying was assessed by “European Cyberbullying Intervention Project Questionnaire” (ECIPQ; [48]). This Likert-type questionnaire comprises 24 items, with five response options according to the frequency which the proposed situations happen (0 = no; 1 = yes, once or twice; 2 = yes, once or twice a month; 3 = yes, around once a week; 4 = yes, more than once a week). This scale assesses both cyber-victimization situations (e.g., “Someone has created a false account to impersonate me”) and cyber-aggression situations (e.g., “I have posted risky photos or videos from another person on the internet”). In this study, the internal consistency was adequate in both the cyber-aggression dimension (ω*_h_* = 0.92; *α* = 0.68) and the cyber-victimization dimension (ω*_h_* = 0.92; *α* = 0.76), as well as for the total scale (ω*_h_* = 0.94; *α* = 0.81).

### 2.3. Procedure

After requesting and obtaining the necessary permission from the management of the secondary education centres, a group of trained researchers moved to the educational centres to distribute the instruments to the selected students. Prior to the data collection, informed consent was obtained from the parents to allow the children to participate in the study. The anonymous and confidential nature of the research was emphasised, and so was the volunteer nature of the participation from the students. Furthermore, the students’ doubts were solved. The average time to fill the questionnaires in ranged from 45 to 60 min.

### 2.4. Data Analysis

First, confirmatory factor analyses (CFA) were conducted using the maximum likelihood estimation method with robust correction (appropriate when the instruments involve ordinal items; [49] to verify the factorial structure of the instruments. The model fit was assessed considering the chi-square significance value of Satorra-Bentler (X^2^S-B) (values higher than 0.01 indicate a good fit). However, because the value of this index depends on other variables such as sample size, other indicators were considered. These included the comparative fit index (CFI), the non-normed fit index (NNFI) (values greater than 0.90 indicate a good fit), and the root mean square error of approximation (RMSEA) (values less than 0.08 indicate a good fit) [50].

The roles of involvement in cyberbullying were established by using the ECIPQ questionnaire. On the basis of the definition established by Smith et al. [4] and bearing in mind the established criteria in other similar studies with the same instrument [39,48], the cyber-victims were identified when they obtained scores higher or equal to 2 (once or twice a month) in any of the cyber-victimization items and scores equal or lower to 1 (once or twice) in all the cyber-aggression items. The cyber-aggressors were those who selected 2 or more (once or twice a month) in any cyber-aggression item and 1 or 0 (once or twice or never) in all the cyber-victimization items. The cyber-bully/victims scored equal or higher to 2 (once or twice a month) in at least any item of the aggression and victimization dimensions. The non-involved were considered those who got a score which was lower or equal to 1 (once or twice) in both cyber-aggression and cyber-victimization items.

To discover the possible differences among the established groups according to the role of involvement in cyberbullying and gender, in their perception of parenting styles and parental discipline, 20 two-way ANOVAS (cyberbullying roles X gender) were carried out. Post hoc contrasts were also carried out by using Bonferroni test. In this analysis will be only commented the main effect of cyberbullying role and the possible interaction with gender, but not the main effect of gender because this last result is not in line with the aim of the study. The adopted significance level to create the measure contrasts was *p* < 0.05. The effect size of the differences between groups was assessed with the statistical Cohen’s *d*. A value of this index lower than 0.50 shows a small effect; values between 0.50 and 0.80 show a moderate effect; values higher than 0.80 show a large effect [51].

To determine the relation between the different dimensions of parenting styles and parental discipline and the involvement in any of the cyberbullying roles established previously, binary logistic regression models were carried out using forward method (wald) for selecting variables. The establishment of “dummy” variables (those which identify if students were or not involved in each of the roles) was carried out by using the same criteria than in the configuration of cyberbullying roles. In this sense, four new dummies variables were created to reflect if the students were or not involved as a cyber-victim (1° dummy variable), cyber-aggressor (2° dummy variable), cyber-bully/victim (3° dummy variable), or if they were or not non-involved (4° dummy variable). Based on the established criteria, for example, in the first dummy variable, students non-categorized as cyber-victim (0) were those who were categorized as cyberbully, cyberbully/victim, and non-involved versus those categorized as victims (1). This analysis was performed three times: one with all the students and the other two, selecting independently girls and boys to verify if there were gender differences in the relation between parenting styles and parental discipline, and the involvement in cyberbullying.

The analyses were carried out using SPSS 18.0 (IBM, Armonk, NY, USA).

## 3. Results

The results concerning the validation of the parenting style questionnaire demonstrated an optimal fit for both the father scale (X^2^S-B = 2631.59; *p* = 0.00; NNFI = 0.98; CFI = 0.98; RMSEA = 0.042) and the mother scale (X^2^S-B = 2680.08; *p* = 0.00; NNFI = 0.98; CFI = 0.98; RMSEA = 0.041), corroborating the original six-factor structure of the scale. The original structure was also verified in the ECIPQ obtaining good fit indices that confirmed the two-factor structure: X^2^S-B = 1009.81; *p* = 0.00; NNFI = 0.93; CFI = 0.93; RMSEA = 0.039. For the Discipline Dimensions Inventory, we first tried the original factor model with second-order factors composed by specific discipline behaviours as first-order factors (for example, the second order factor “Inductive Discipline” was formed by three first-order factors: Diversion, Explain/Teach, and Rewarding), and obtained inadequate fits for the mother scale (X^2^S-B = 4292.33; *p* = 0.00; NNFI = 0.53; CFI = 0.58; RMSEA = 0.095), and the father scale (X^2^S-B = 4111.03; *p* = 0.00; NNFI = 0.50; CFI = 0.56; RMSEA = 0.095). For this reason, then we proceeded to test the other factorial structure suggested by the authors of the questionnaire: a first-order structure composed by the main four discipline procedures. Optimal results were achieved for both the father scale (X^2^S-B = 2578.72; *p* = 0.00; NNFI = 0.95; CFI = 0.96; RMSEA = 0.073) and the mother scale (X^2^S-B = 2748.22; *p* = 0.00; NNFI = 0.95; CFI = 0.95; RMSEA = 0.074). As literature has shown that some parental practices are related to each other [20,25,40] the Spearman’s correlation between all parenting practices variables were calculated. Affection and communication, promotion of autonomy, behavioural control, humour, disclosure and inductive discipline showed a significant and positive relationship between them. In the same way, behavioural and psychological control, punishment, response cost, and supervision showed a direct relationship between them. One exception was the relationship between behavioural control and punishment which was inverse. Other inverse relationships were those established between affection and communication, promotion of autonomy, humour, disclosure, and all of the discipline procedures except inductive discipline. Affection and communication, promotion of autonomy, humour, and disclosure also showed negative relationships with psychological control (see Table 1). These findings were in line with those found in previous studies [25,47,52].

### 3.1. Description of Parenting Practices According the Roles of Cyberbullying and Gender

Prevalence of cyberbullying involvement was calculated: 19% of students were involved in cyberbullying (6.6% cyber-victims, 4.2% cyber-aggressors, 8.2% cyber-bully/victims) and 81% were not involved.

Two-way ANOVAS, which were performed to examine the relation between the maternal parenting practices and the roles of cyberbullying and the possible interaction between them and gender, showed statistically significant differences among the groups—non-involved, cyber-victims, cyber-aggressors, and cyber-bully/victims—in all the maternal parenting practices (affection and communication, behavioural and psychological control, promotion of autonomy, humour, disclosure, inductive discipline, punishment, response cost, and supervision) (see Table 2). The interaction between gender and cyberbullying roles was found to have a significant effect on the affection and communication, psychological control, inductive discipline, punishment and response cost (see Table 3). However, an effect of the interaction between gender and cyberbullying roles in behavioural control (F_(3, 1821)_ = 1.41; *p* = 0.238), promotion of autonomy (F_(3, 1815)_ = 1.75; *p* = 0.155), humour (F_(3, 1804)_ = 1.28; *p* = 0.277), disclosure (F_(3, 1745)_ = 1.34; *p* = 0.258), and supervision (F_(3, 1779)_ = 1.85; *p* = 0.136) was not found. 

In the univariate analysis, post hoc tests showed that non-involved students were those who presented the highest scores in the following dimensions: affection and communication, promotion of autonomy, and humour. However, they showed the lowest scores regarding the parental use of techniques related to punitive discipline, such as the physical or psychological punishment and response cost, in comparison to those categorised as cyber-bully/victims, cyber-aggressors and cyber-victims. On the other hand, differences were found among non-involved, cyber-aggressors and cyber-bully/victims regarding the practice of behavioural control and the disclosure behaviour, the last two roles showed the lowest score in both cases. Additionally, cyber-victims showed a higher perception of behavioural control in comparison to cyber-aggressors, and a greater perception of psychological control than non-involved, their score in this last variable was very similar to cyber-bully/victims. Furthermore, significant differences were observed between these last students and the non-involved in the dimension of inductive discipline, being the cyber-bully/victims’ scores higher. Finally, the students who were cyber-victims and cyber-bully/victims showed a higher parental use of supervision, in comparison to the non-involved. Differences between the first roles were also found, showing cyber-bully/victims the greatest perception of supervision. The effect sizes of the differences were small and moderate in all the cases (see Table 2).

**Figure 1:**
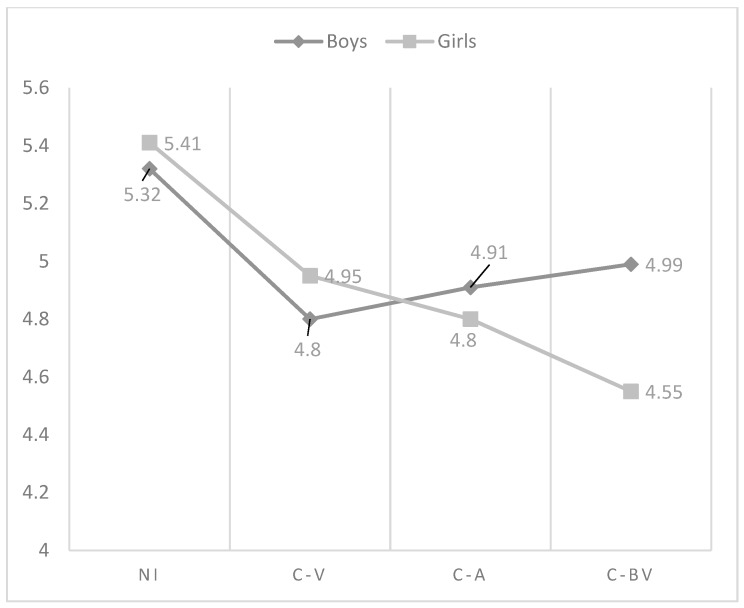
Effect of the interaction of gender and cyberbullying roles on mother affection and communication. Notes: NI = Non-involved; C-V = Cyber-victim; C-A = Cyber-Aggressor; C-BV = Cyber-bully/victim.

**Figure 2:**
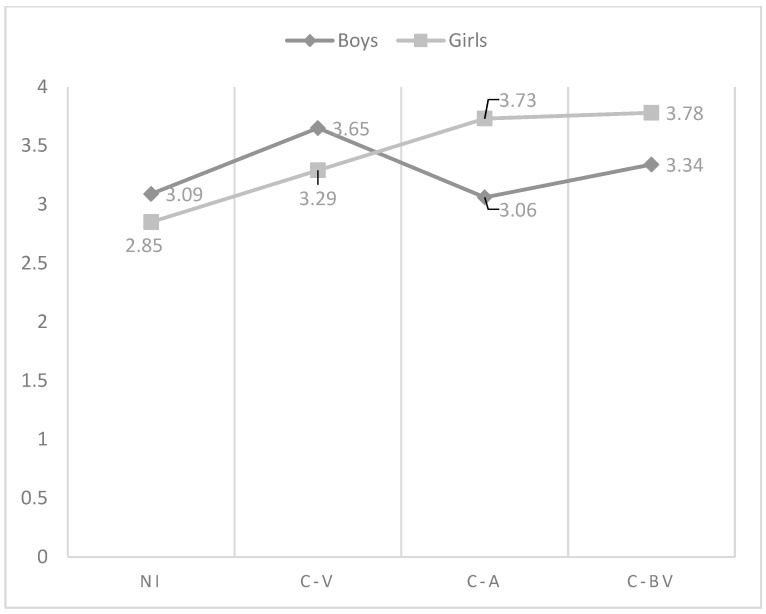
Effect of the interaction of gender and cyberbullying roles on mother psychological control. Notes: NI = Non-involved; C-V = Cyber-victim; C-A = Cyber-Aggressor; C-BV = Cyber-bully/victim.

**Figure 3:**
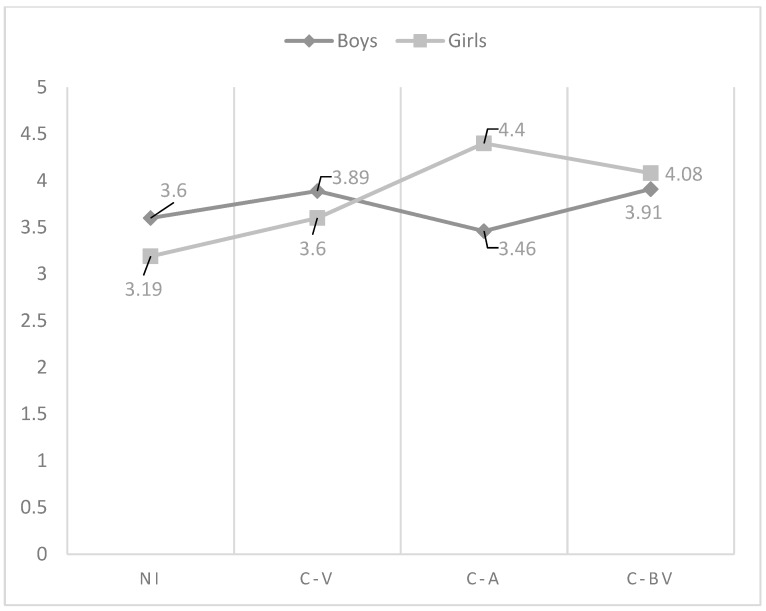
Effect of the interaction of gender and cyberbullying roles on mother inductive discipline. Notes: NI = Non-involved; C-V = Cyber-victim; C-A = Cyber-Aggressor; C-BV = Cyber-bully/victim.

**Figure 4:**
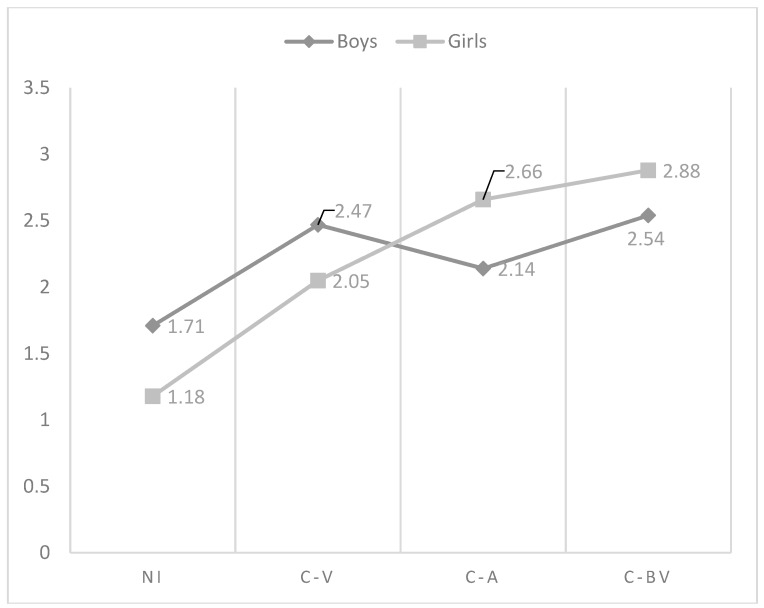
Effect of the interaction of gender and cyberbullying roles on mother punishment. Notes: NI = Non-involved; C-V = Cyber-victim; C-A = Cyber-Aggressor; C-BV = Cyber-bully/victim.

**Figure 5:**
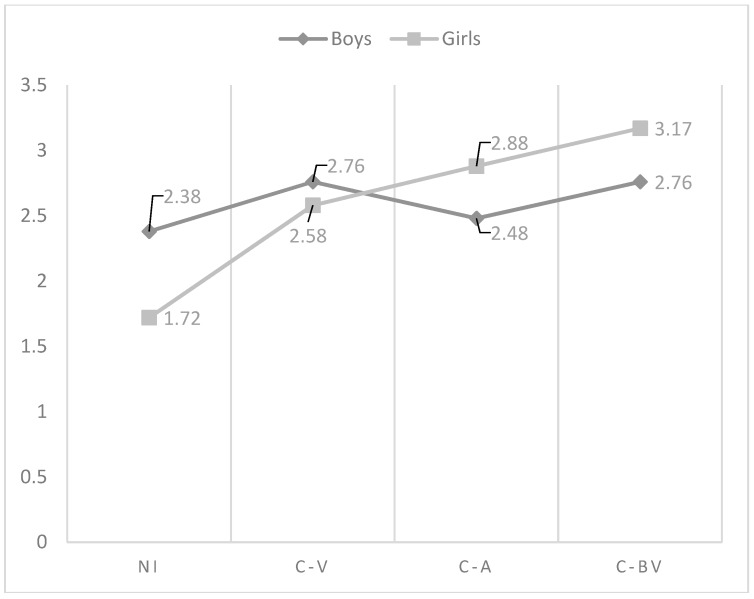
Effect of the interaction of gender and cyberbullying roles on mother response cost. Notes: NI = Non-involved; C-V = Cyber-victim; C-A = Cyber-Aggressor; C-BV = Cyber-bully/victim.

As regards the effect of the interaction between gender and role of cyberbullying in the affection and communication dimension, the analysis indicated that the highest perception was found in non-involved in boys and girls, but while in boys cyber-victims were those who reflect the lowest perception of this dimension, in girls were cyber-bully/victims, followed of cyber-aggressors (see Figure 1). With respect to psychological control, in boys, non-involved showed the lowest perception, being victims who reflected the highest scores. Non-involved girls showed also the lowest perception of psychological control. However, in this last group, were cyber-bully/victims who got the highest scores (see Figure 2). Similarly, non-involved girls indicated the lowest use of inductive discipline by their mother, finding the highest values in cyber-bully/victims boys (see Figure 3). However, there were no significant differences found between the cyberbullying roles comparing boys and girls to themselves. In punishment and response costs the highest scores were obtained by those boys categorized as cyber-victims, who showed similar values compared to cyber-bully/victims. Non-involved showed the lowest scores. However, the differences between non-involved and cyber-bully/victims were significant only in punishment. In girls, cyber-bully/victims obtained the highest scores, followed by cyber-aggressors and cyber-victims, respectively (see Figure 4 and Figure 5). However, the differences between non-involved and cyber-aggressors were not significant in response cost. Two-way ANOVA about the differences in mother parenting practices according to the interaction between the role of involvement in cyberbullying and gender can be seen in Table 3. This table has included only those variables significantly affected by the interaction between gender and cyberbullying roles.

When analysing the differences between the paternal parenting practices, and the roles of cyberbullying, the results from the ANOVA test (see Table 4) showed the existence of significant statistical differences among the established groups in all the educational practices. The interaction between gender and cyberbullying roles was found to have a significant effect on psychological control and promotion of autonomy (see Table 5). However, it was not found an effect of the interaction between gender and cyberbullying roles in affection and communication (F_(3, 1787)_ = 1.21; *p* = 0.305), behavioural control (F_(3, 1748)_ = 0.63; *p* = 0.594), humour (F_(3, 1804)_ = 1.28; *p* = 0.277), disclosure (F_(3, 1674)_ = 0.97; *p* = 0.404), inductive discipline (F_(3, 1668)_ = 2.39; *p* = 0.067), punishment (F_(3, 1611)_ = 1.07; *p* = 0.358), response cost (F_(3, 1650)_ = 2.16; *p* = 0.090), and supervision (F_(3, 1690)_ = 1.72; *p* = 0.160).

Particularly, non-involved adolescents were those who got higher scores in the dimensions of affection and communication, humour and disclosure, and lower scores in the procedures of punishment and response cost, in comparison to the adolescents categorised as cyber-bully/victims, cyber-aggressors and cyber-victims, as the analysis showed afterwards. Cyber-victims and cyber-bully/victims showed a greater perception of psychological control than those non-involved and indicated that their progenitors did not tend to promote their autonomy. Cyber-aggressors and cyber-bully/victims reflected the lowest scores in behavioural control; however, they showed a greater use of supervision as a procedure used by parents to manage discipline, establishing significant differences with the non-involved students. Finally, significant differences were observed between the last students and the cyber-bully/victims in the dimension of inductive discipline, being lower the scores from the non-involved students. The effect sizes on the differences were small and moderate in all the cases (see Table 4).

**Figure 6:**
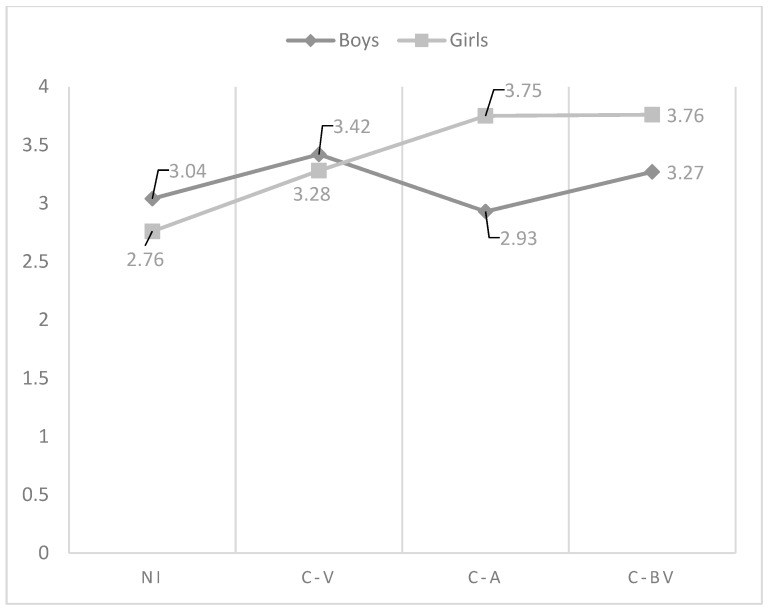
Effect of the interaction of gender and cyberbullying roles on father psychological control. Notes: NI = Non-involved; C-V = Cyber-victim; C-A = Cyber-Aggressor; C-BV = Cyber-bully/victim.

**Figure 7:**
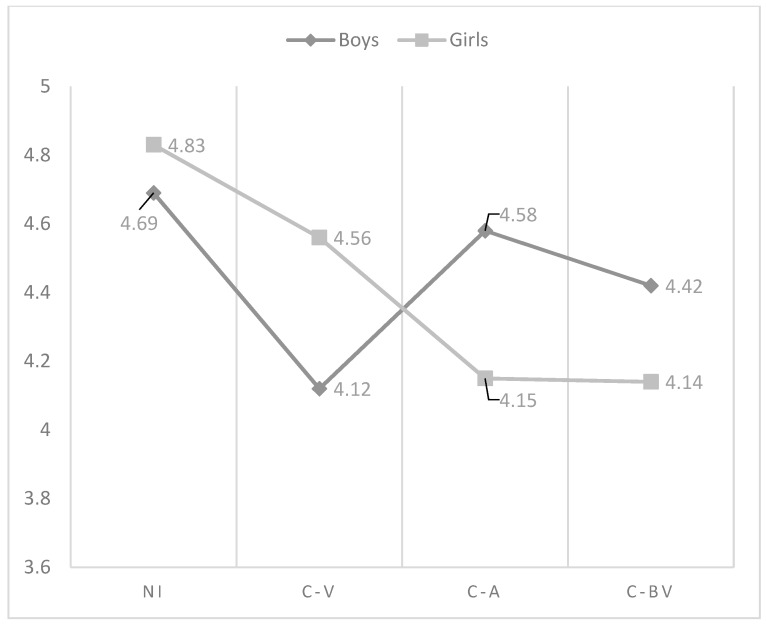
Effect of the interaction of gender and cyberbullying roles on father promotion of autonomy. Notes: NI = Non-involved; C-V = Cyber-victim; C-A = Cyber-Aggressor; C-BV = Cyber-bully/victim.

As regards the effect of the interaction in psychological control, in boys there were no differences in the perception of this practice according to the role of involvement in cyberbullying. However, non-involved girls showed the lowest perception of psychological control compared to cyber-aggressors and cyber-bully/victims (see Figure 6). With respect to promotion of autonomy, cyber-bully/victim girls had the lowest scores compared to non-involved. However, in the case of boys, the students who showed the lowest perception of promotion of autonomy were cyber-victims. The scores of cyber-aggressors and non-involved were very similar, being the highest (see Figure 7). Two-way ANOVAS about the differences in father parenting practices according to the interaction between the role of involvement in cyberbullying and gender can be seen in Table 5. In this table have been only included those variables affected significantly by the interaction between gender and cyberbullying roles.

### 3.2. Relation between Parenting Practices and the Involvement in the Different Cyberbullying Roles

The binary logistic regression analysis, which used as a dependent variable the role of cyber-victim, offered a correct estimation (*χ*^2^ = 6.06, *p* < 0.01) in the 92.9% of the cases for the general sample. In the case of boys, the model correctly estimated the 93.4% of the cases (*χ*^2^ = 10.75, *p* < 0.01), with a slightly lower percentage to the model with only girls (92.4%; *χ*^2^ = 11.19, *p* ≤ 0.01). The variables which took part in the equation in each case can be observed in Table 6. Specifically, affection and communication of father and mother had an inverse effect on involvement as a cyber-victim (from mother in case of boys and from father and mother in general sample). Additionally, mother’s punishment had a direct effect on this involvement in girls. Overall, those variables achieved to explain 5.5% of the variance of the dependent variable when we worked with the total sample, and 5.2% and 4.7% when we selected only boys and only girls, respectively.

The binary logistic regression analysis carried out for the cyber-aggressor role provided us with a correct estimation of the 95.9% in the cases for the general sample (*χ*^2^ = 8.34, *p* < 0.05). In the case of boys, the model correctly estimated the 94.4% of the cases (*χ*^2^ = 6.3, *p* < 0.05), a slightly lower percentage to that the model with only girls (97.4%; *χ*^2^ = 7.59, *p* < 0.01). The variables which took part in the equation in each case can be observed in Table 6. Affection and communication of father had an inverse effect on involvement as cyber-aggressor in all the subsamples. However, father’s promotion of autonomy and father and mother inductive discipline had a direct effect; in the first case only for boys, and in the second, in the general sample and in girls, respectively. Taking into account the odds ratio, the variables which seem to explain better the involvement as a cyber-aggressor was inductive discipline of father and mother for the general sample and in the case of girls respectively, and father’s promotion of autonomy in the case of boys. Overall, those variables achieved to explain 5.9% of the variance in the dependent variable when we worked with the total sample, and 6.2% and 19.4% when we selected only boys and only girls, respectively.

The binary logistic regression analysis carried out for the cyber-bully/victim role provided us with a correct estimation of 92.3% in the cases for the general sample (*χ*^2^ = 4.01, *p* < 0.05). In the case of boys, the model correctly estimated 87.8% of the cases (*χ*^2^ = 8.47, *p* < 0.001), a lower percentage to the model with only girls (96.5%; *χ*^2^ = 19.9, *p* < 0.01). The variables which took part in the equation in each case can be observed in Table 6. Disclosure to mother and father had an inverse effect on involvement as a cyber-bully/victim in the general sample and boys, specifically. However, father’s supervision (in the general sample and boys) and mother’s punishment (in girls) had a direct effect. Taking into account the odds ratio, the variables which seem to explain better the involvement as a cyber-bully/victim was mother’s punishment and father’s supervision in the general sample and father’s supervision in the case of boys. Overall, those variables achieved to explain 8.6% of the variance in the dependent variable when we worked with the total sample, and 6.9% and 13.1% when we selected only boys and only girls, respectively.

The binary logistic regression analysis which used as a dependent variable the role of non-involved offered a correct estimation (*χ*^2^ = 5.77, *p* < 0.05) in 81.1% of the cases for the general sample. In the case of boys, the model correctly estimated 75.6% of the cases (*χ*^2^ = 7.31, *p* < 0.01), with a higher percentage to the one we found by selecting only girls (86.3%; *χ*^2^ = 7.88, *p* ≤ 0.01). Those variables which took part in the equation in each case can be observed in Table 6. Specifically, affection and communication of father and mother had an inverse effect on involvement as a cyber-victim (from mother in case of boys and from father and in general sample). Additionally, mother’s punishment had a direct effect on this involvement in girls and the general sample, and mother’s response cost and supervision and father’s inductive discipline had the same effect on girls, boys, and in the general sample, respectively. Taking into account the odds ratio, the variables which seem to explain better the non-involvement in cyberbullying was affection and communication, from the father for the general sample and girls, and from the mother in the case of boys. Overall, those variables achieved to explain 11.8% of the variance of the dependent variable when we worked with the total sample, and 9.9% and 16.1% when we selected only boys and only girls, respectively.

## 4. Discussion

This study was carried out to explore the possible relation between parenting practices and the involvement in cyberbullying taking into account the gender of teenagers. The first aim was directed to describe parental practices of different roles. In particular, students non-involved in cyberbullying -compared to those involved- perceived a greater affection and communication, a better humour from their progenitors, as well as a higher promotion of their autonomy by their mother, presenting a more frequent disclosure behaviour towards the father. Those involved, cyber-victims, cyber-aggressors and cyber-bully/victims, stated experiencing a greater use of physical punishment or psychological aggression by their progenitors as well as being object of disciplinary procedures, such as the response cost and the supervision by the father. These results are consistent with the first hypothesis and the previous evidence, in which differences in the mother’s and father’s educational practices regarding involvement in cyberbullying are not specified. In this sense, previous research also emphasizes the positive effect of affection and communication and the promotion of autonomy, as well as the risks under the use of punitive or excessive discipline regarding the involvement in this violent phenomenon [22,32,36,38]. With regard to parental humour, although it may seem that this dimension has not been analysed in relation to cyberbullying, the observed trend coincides with bullying studies, which place it as a clearly beneficial attitude for minors, and which is, in any case, associated with a lower involvement in violent dynamics [39,40,41].

It was observed that minors who were cyber-victims or cyber-bully/victims tend to perceive more psychological control from both progenitors, a greater use of discipline strategies, such as response cost and supervision by the mother, and a lower promotion of paternal autonomy. There are only a few studies that have examined the importance of promoting minors’ autonomy and there are even less studies that have used involvement in cyberbullying as a dependent variable. In general, it has been observed that this practice stimulates the acquisition of essential competences to be able to successfully manage future difficulties and, therefore, to make decisions on their own lives with independence and conviction [53]. On the contrary, a parental attitude that limits the filial autonomy makes easy the minors’ dependence feeling towards their parents when they have to face problematic situations. This dependence would make difficult the successful resolution of problematic situations such as cyberbullying [6,39]. Similarly, other practices, such as the psychological control—more used by cyber-victims’ and cyber-bully/victims’ progenitors—have clearly shown their negative effect on minors’ adjustment, promoting inefficacy and low self-esteem feelings as well as lessening their the capacity to develop a successful defence against an unjustified aggressive act at the hands of another peer, regardless the means in which the aggression takes place [38,40,42].

Being involved as cyber-aggressors or cyber-bully/victims has shown to be associated with a lower disclosure towards both progenitors, something to be expected given the intention of concealing the immoral action undertaken to avoid its negative consequences [36,37]. Nevertheless, disclosure towards the father was also a less widespread practice among the victims. This result could be interpreted from diverse viewpoints. It could be due to the fact that the father is perceived by the cyber-victims as a lower source of assistance. This perception would coincide with what we found on other samples with adolescents that pointed at the mother as a closer figure, who rouses a greater trust or who reflects a greater ability to stimulate this subtle control technique in her children [25]. On the other hand, this result also seems to suggest that, even though it is more common to resort to the mother to disclose personal information, its possible protective effect regarding cyberbullying would only occur when the father becomes an interlocutor in this regard. Therefore, the use of disclosure with both progenitors could be showing that there is a positive and trustworthy environment among all the members of that family, what could favour the adjustment and welfare of all of them [27,29].

The cyber-aggressors and cyber-bully/victims have also shown to perceive a lower behavioural control and a greater use of discipline procedures like the response cost and the supervision. This result seems to evidence the necessity of promoting parental monitoring, not only by developing norms related to the minor’s general behaviour, but also by using the procedures of parental mediation, which allow to know, and even to limit, the use that young people make of their mobile phones and computers, guiding them to avoid the risks and extract the potentiality these means may have [14]. Therefore, it would be advisable to use inducement, based on reasoning and stimulation of the moral power and empathy [48]. However, the exclusive use of this technique neither seems to give good results. The results show that cyber-bully/victims, which are the most complex and maladjusted role of the phenomenon, indicated a more frequent use of this discipline procedure by their progenitors than the rest of the people involved. This could be indicating that maybe students involved in cyberbullying are difficult sons and daughters whose parents have to use different discipline techniques to correct their misbehaviour compared to those not involved in this violent dynamic, but also, that inductive discipline is not so positive if it is not accompanied by other protective attitudes, such as empathy and respect by the adolescent as some recent informative perspectives suggest, such as positive discipline [54]. In any case, the effect of this discipline perspective has not been empirically examined, so it would be necessary to study its effects applied to cyberbullying involvement.

As it was stated in the second hypothesis, an effect of the interaction between gender and cyberbullying roles in some parental practices was found. The parenting practices affected by this effect were maternal affection and communication and psychological control, paternal promotion of autonomy and psychological control, and the use of different discipline procedures by the mother, such as inductive discipline, punishment and response cost. In general, it can be observed that, in boys, the most negative results in each dimension were found in cyber-victims or cyber-bully/victims, while, in girls, they were associated to the role of cyber-bully/victim or cyber-aggressor. In this sense, boys cyber-victims obtained the lowest scores on maternal affection and communication and paternal promotion of autonomy, and the highest on paternal psychological control. In addition, boys cyber-victims and cyber-bully/victims showed the greatest perception of maternal psychological control and the most frequent use of the different discipline procedures. These same results were found in the case of girls in cyber-bully/victims or cyber-aggressors. Although previous research has shown that negative parental behaviours tend to affect to boys and girls in a different way, to the best of our knowledge, it is the first study that reports these results applied to cyberbullying. In this sense, recent studies have reported that negative parenting (especially those parental practices related to the management of misbehaviour such as high control or punishment) increases the likelihood of girls to develop externalizing and aggressive behaviours [55,56] and that girls seem to be more affected by negative parental practices which favour that they feel more hurt and less loved than boys [57]. This evidence helps to understand our findings which suggest that negative parenting in girls is associated not only to the lack of skills to cope cyber-victimization situations, but also to an important maladjustment which also leads them to bully others through ICT. However, it is necessary to continue studying gender differences in the relationship between parenting practices and cyberbullying involvement through longitudinal studies to be able to get specific conclusions for taking into account in the design of interventions to prevent cyberbullying.

The second aim was to know the predictive value of parenting practices, on the involvement in different roles of cyberbullying, paying attention to the progenitors’ and minors’ gender. The role that parenting practices play does not seem to be exactly the same for boys and for girls involved in cyberbullying. Affection and communication dimension was an apparent protective factor for the involvement as a cyber-victim (especially in boys) and cyber-aggressor, which was also positive and strongly related to the non-involved role. In addition, it is important to underline the importance of disclosure towards the father and the promotion of autonomy of father to prevent the involvement of boys as a cyber-bully/victim and cyber-aggressor respectively. On the other side, the use of the different discipline procedures, and especially punishment for girls, were the parental behaviours that suppose a greater risk for the involvement as a cyber-victim, cyber-aggressor and cyber-bully/victim, showing these same procedures a negative relationship with the role of non-involved.

These results underline, as the third hypothesis indicated, the importance of affection and communication as a protective parental attitude in the involvement in cyberbullying, agreeing with previous studies on aggression [40,43]. Moreover, findings suggest the relevance of coercive practices to explain the adolescent involvement in this violent phenomenon. Although it is difficult to identify a specific factor which contribute to the involvement of boys, it seems that positive paternal attitudes, such as promoting autonomy and others which favour the disclosure of adolescents to the father are very important for this group. Girls, however, did not show any protective factor beyond affection and communication, but the results could be indicating their special vulnerability to punitive discipline, such as physical and psychological punishment, which might increase the risk to become an object of online maltreatment or develop also aggressive acts turning into cyber-bully/victim. Previous studies have also highlighted the negative effect of punishment in psychosocial adjustment of adolescents, and especially girls [56] and the positive effect of promoting teenagers’ autonomy and stimulate their self-disclosure [41]. Nevertheless, more research is necessary to be able to explain this gender differences in the protective or risk effect of parenting styles in cyberbullying involvement.

Finally, as stated in the fourth hypothesis, girls seem to be those who suffered more impact by the family influence, which could explain the parenting styles and the parental discipline in girls reaching up to a 19.4% of the probability of being involved in cyberbullying, while this percentage is reduced to 9.9% in the case of boys. The different socialization that minors receive following their gender could explain this distribution of risk factors and parental protection regarding cyberbullying and its differential impact in boys and girls. Therefore, it is advisable to pay attention to this fact and standardize the practices, attitudes and values that progenitors transmit to their children, as their influence goes beyond preventing or stimulating their involvement in violent dynamics, such as cyberbullying, conditioning their future development paths [25,43]. In any case, it would be advisable to further study gender differences in the relationship between parenting practices and the involvement in cyberbullying to extract clearer tendencies which are useful from a preventive point of view.

This study presents some limitations regarding its design and the way of measuring the construct of interest. This way, because of the use of self-report measures to collect information, we must be careful when interpreting the results, as these instruments measure the subjective perception from the surveyed adolescents regarding parenting styles and their progenitors’ discipline, and they could entail certain bias, like social desirability or acquiescence. Nevertheless, the adolescents’ response seems to be the most reliable source of information since they are less influenced by bias when describing their parents’ educational styles who, unlike their progenitors, usually search for social acceptance [38]. The transversal design of the study also limits the possibility of drawing consistent conclusions on the accurate relation between parenting styles and parental discipline and the adolescents’ involvement in cyberbullying, so we must interpret the data with certain prudence, avoiding establishing cause-effect relations.

## 5. Conclusions

The results of this study indicate that parenting practices are important factors to be taken into consideration regarding adolescents’ involvement in cyberbullying, especially in girls. Therefore, the development of intervention initiatives whose purpose is fighting against cyberbullying must include family education to improve the awareness on the importance that the relationship with their children has for their welfare [58]. The results of this research seem to reflect that this style coincides with what has been described as democratic supervisor or indulgent, depending on the research and the questionnaire used to assess parenting style [21,41]. This style makes it easy to establish a relaxed family environment, by fostering affection and communication, whilst it promotes minors’ autonomy and good humour at home. To manage discipline, induction, reasoning, and parental monitoring are useful, since discipline is based on the establishment of norms and limits necessary to foster minor’s regulation; additionally, it avoids other coercive practices, such as physical punishment or psychological control. In any case, the best parenting style depends on the psychosocial adjustment measure studied and also on the culture. In this sense, although in Spain and, in general, in western society, the styles which include these parenting practices seem to be better [41,59,60], maybe in other cultures and regarding different adjustment measures from cyberbullying the best for children and adolescents could be an adaptation of these behaviours to their own culture. For example, in eastern countries more restrictive parenting practices, such as higher monitoring and less promotion of autonomy, seem to be associated with good results [61]. Taking this into account, and focusing on the aims of this study, it would be interesting to examine the effect of parental practices, specifically those which seek to limit and monitor the use of ICT, such as parental mediation [14] on cyberbullying involvement of teenagers from western and eastern countries.

## Figures and Tables

**Table 1:** Spearman’s correlation between parenting practices variables.

	1	2	3	4	5	6	7	8	9	10	11	12	13	14	15	16	17	18	19	20
A-M (1)	1.000	0.267 **	−0.293 **	0.603 **	0.656 **	0.557 **	0.054 +	−0.394 **	−0.141 **	−0.203 **										
BC-M (2)		1.000	0.186 **	0.261 **	0.215 **	0.391 **	0.214 **	−0.058 +	0.154 **	0.071 *										
PC-M (3)			1.000	−0.272 **	−0.291 **	−0.165 **	0.265 **	0.491 **	0.392 **	0.399 **										
PA-M (4)				1.000	0.582 **	0.499 **	0.038	−0.354 **	−0.143 **	−0.170 **										
H-M (5)					1.000	0.531 **	0.051 +	−0.415 **	−0.157 **	−0.203 **										
D-M (6)						1.000	0.069 *	−0.327 **	−0.118 **	−0.140 **										
ID-M (7)							1.000	0.413 **	0.634 **	0.537 **										
P-M (8)								1.000	0.593 **	0.582 **										
RC-M (9)									1.000	0.618 **										
S-M (10)										1.000										
A-F (11)	0.647 **	0.229 **	−0.235 **	0.453 **	0.473 **	0.401 **	0.049 +	−0.337 **	−0.126 **	−0.153 **	1.000									
BC-F (12)	0.265 **	0.783 **	0.076 *	0.267 **	0.246 **	0.374 **	0.152 **	−0.129 **	0.074 *	0.014	0.386 **	1.000								
FC-F (13)	−0.210 **	0.146 **	0.810 **	−0.178 **	−0.171 **	−0.111 **	0.260 **	0.398 **	0.341 **	0.351 **	−0.237 **	0.151 **	1.000							
FA-F (14)	0.472 **	0.247 **	−0.238 **	0.810 **	0.456 **	0.398 **	0.017	−0.307 **	−0.134 **	−0.135 **	0.605 **	0.353 **	−0.238 **	1.000						
H-F (15)	0.656 **	0.215 **	−0.291 **	0.582 **	10.000 **	0.531 **	0.051 +	−0.415 **	−0.157 **	−0.203 **	0.473 **	0.246 **	−0.171 **	0.456 **	1.000					
D-F (16)	0.409 **	0.336 **	−0.130 **	0.382 **	0.389 **	0.740 **	0.053 *	−0.294 **	−0.091 **	−0.102 **	0.621 **	0.455 **	−0.136 **	0.513 **	0.389 **	1.000				
ID-F (17)	0.077 *	0.206 **	0.228 **	0.061 +	0.076 +	0.074 *	0.920 **	0.360 **	0.586 **	0.504 **	0.128 **	0.222 **	0.274 **	0.090 **	0.076 +	0.145 **	1.000			
P-F (18)	−0.304 **	−0.077 *	0.380 **	−0.286 **	−0.300 **	−0.260 **	0.415 **	0.862 **	0.549 **	0.524 **	−0.324 **	−0.106 **	0.460 **	−0.315 **	−0.300 **	−0.283 **	0.417 **	1.000		
RC-F (19)	−0.104 **	0.130 **	0.328 **	−0.097 **	−0.101 **	−0.092 **	0.598 **	0.512 **	0.901 **	0.557 **	−0.075 *	0.131 **	0.380 **	−0.097 **	−0.101 **	−0.037	0.628 **	0.590 **	1.000	
S-F (20)	−0.162 **	0.042	0.336 **	−0.130 **	−0.151 **	−0.107 **	0.527 **	0.503 **	0.579 **	0.915 **	−0.122 **	0.044	0.381 **	−0.111 **	−0.151 **	−0.073 *	0.547 **	0.553 **	0.613 **	1.000

Notes: A = Affection and Communication; BC = Behavioural Control; PC = Psychological Control; PA = Promotion of Autonomy; H = Humour; D = Disclosure; ID = Inductive Discipline; P = Punishment; RC = Response Cost; S = Supervision; M = Mother; F = Father; + = *p* < 0.05; * *p* < 0.01; ** *p* < 0.001.

**Table 2:** ANOVA to determine the differences in parenting practices of mother according to the role of involvement in cyberbullying.

	Groups	*N*	Mean	SD	F	F.D.	Sig.	Post Hoc	Cohen’s *d*
Affection and Communication	NI	1515	5.37	0.83	26.49	(3, 1867)	0.00	NI ≠ VIC	0.55
	VIC	124	4.89	1.34				NI ≠ AGGR	0.57
	AGGR	79	4.89	1.08				NI≠ B-VIC	0.55
	B-VIC	150	4.90	1.13					
Behavioural Control	NI	1481	4.78	1.12	6.74	(3, 1827)	0.00	NI ≠ AGGR	0.43
	VIC	122	4.74	1.09				NI ≠ B-VIC	0.25
	AGGR	79	4.30	1.25				VIC ≠ AGGR	0.38
	B-VIC	146	4.50	1.19					
Psychological Control	NI	1461	2.97	1.23	12.04	(3, 1808)	0.00	NI ≠ VIC	0.39
	VIC	124	3.46	1.27				NI ≠ B-VIC	0.38
	AGGR	77	3.23	1.21					
	B-VIC	147	3.44	1.19					
Promotion of Autonomy	NI	1476	4.91	0.97	15.98	(3, 1821)	0.00	NI ≠ VIC	0.33
	VIC	121	4.58	1.21				NI ≠ AGGR	0.41
	AGGR	79	4.51	1.26				NI ≠ B-VIC	0.49
	B-VIC	146	4.43	1.16					
Humour	NI	1472	5.00	0.99	21.49	(3, 1810)	0.00	NI ≠ VIC	0.39
	VIC	121	4.60	1.22				NI ≠ AGGR	0.46
	AGGR	74	4.54	1.18				NI ≠ B-VIC	0.58
	B-VIC	144	4.41	1.21					
Disclosure	NI	1422	4.55	1.27	15.69	(3, 1750)	0.00	NI ≠ AGGR	0.51
	VIC	114	4.24	1.50				NI ≠ B-VIC	0.49
	AGGR	74	3.90	1.35					
	B-VIC	141	3.93	1.32					
Inductive discipline	NI	1430	3.39	1.72	5.94	(3, 1756)	0.00	NI ≠ B-VIC	0.33
	VIC	117	3.74	1.87					
	AGGR	74	3.72	1.79					
	B-VIC	136	3.95	1.68					
Punishment	NI	1379	1.43	1.60	30.35	(3, 1700)	0.00	NI ≠ VIC	0.50
	VIC	120	2.26	2.21				NI ≠ AGGR	0.53
	AGGR	71	2.29	1.82				NI ≠ B-VIC	0.73
	B-VIC	131	2.62	2.03					
Response Cost	NI	1425	2.03	1.76	13.86	(3, 1759)	0.00	NI ≠ VIC	0.35
	VIC	120	2.67	2.28				NI ≠ B-VIC	0.46
	AGGR	76	2.59	1.89				NI ≠ AGGR	0.31
	B-VIC	139	2.85	1.85					
Supervision	NI	1444	1.69	1.78	23.24	(3, 1785)	0.00	NI ≠ VIC	0.33
	VIC	122	2.28	2.12				NI ≠ B-VIC	0.64
	AGGR	74	2.22	1.89				B-VIC ≠ VIC	0.30
	B-VIC	146	2.94	2.28					

Notes: NI = Non-involved; VIC = Cyber-victim; AGGR = Cyber-aggressor; B-VIC = Cyber-bully/Victim; F.D. = Freedom Degrees; SD = Standard Deviation.

**Table 3:** Two-way ANOVA about the differences in mother parenting practices according to the interaction between the role of involvement in cyberbullying and gender.

Variable	Group	Sex	Mean	SD	*N*	F	F.D.	Sig.	Post Hoc	Cohen’s *d*
Affection and communication	NI	boys	5.31	0.85	710	2.94	(3, 1863)	0.032	NI 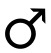 ≠ VIC 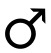	−0.56
		girls	5.41	0.80	801				NI 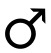 ≠ AGGR 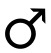	−0.45
	VIC	boys	4.80	1.30	63				NI 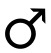 ≠ B-VIC 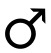	−0.36
		girls	4.95	1.37	61				NI 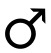 ≠ B-VIC 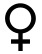	−0.86
	AGGR	boys	4.91	1.12	59				NI 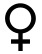 ≠ VIC 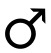	−0.72
		girls	4.80	0.94	20				NI 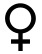 ≠ VIC 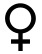	−0.54
	B-VIC	boys	4.99	1.02	118				NI 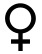 ≠ AGGR 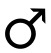	−0.60
		girls	4.55	1.42	32				NI 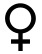 ≠ B-VIC 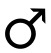 NI 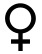 ≠ B-VIC 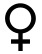	−0.50 −1.03
Psychological control	NI	boys	3.09	1.24	680	4.98	(3, 1802)	0.002	NI 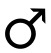 ≠ NI 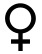	−0.19
		girls	2.85	1.20	776				NI 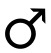 ≠ VIC 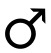	0.45
	VIC	boys	3.65	1.18	62				NI 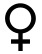 ≠ VIC 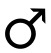	0.66
		girls	3.29	1.34	61				NI 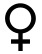 ≠ B-VIC 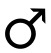	0.41
	AGGR	boys	3.06	1.22	58				NI 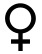 ≠ B-VIC 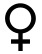	0.77
		girls	3.73	1.05	19					
	B-VIC	boys	3.34	1.15	116					
		girls	3.78	1.29	31					
Inductive discipline	NI	boys	3.60	1.79	659	3.55	(3, 1750)	0.014	NI 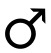 ≠ NI 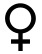	−0.79
		girls	3.19	1.62	766				NI 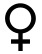 ≠ B-VIC 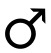	−0.44
	VIC	boys	3.89	2.07	58					
		girls	3.60	1.65	58					
	AGGR	boys	3.46	1.66	54					
		girls	4.40	1.95	20					
	B-VIC	boys	3.91	1.75	109					
		girls	4.08	1.36	27					
Punishment	NI	boys	1.70	1.88	622	3.30	(3, 1694)	0.019	NI 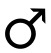 ≠ NI 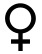	−0.33
		girls	1.18	1.27	752				NI 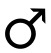 ≠ VIC 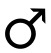	0.40
	VIC	boys	2.47	2.22	61				NI 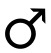 ≠ B-VIC 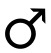	0.44
		girls	2.05	2.20	58				NI 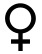 ≠ VIC 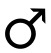	0.94
	AGGR	boys	2.14	1.65	52				NI 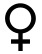 ≠ VIC 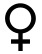	0.64
		girls	2.66	2.20	19				NI 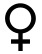 ≠ AGGR 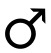	0.74
	B-VIC	boys	2.54	1.87	104				NI 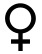 ≠ AGGR 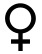	1.13
		girls	2.88	2.56	27				NI 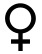 ≠ BVIC 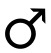 NI 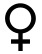 ≠ BVIC 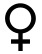	1.00 1.27
Response cost	NI	boys	2.38	1.95	657	4.45	(3, 1754)	0.004	NI 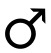 ≠ NI 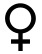	−0.38
		girls	1.72	1.51	763				NI 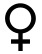 ≠ VIC 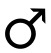	0.19
	VIC	boys	2.76	2.37	60				NI 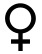 ≠ VIC 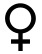	0.10
		girls	2.58	2.19	60				NI 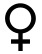 ≠ BVIC 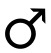	0.19
	AGGR	boys	2.48	1.65	57				NI 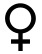 ≠ BVIC 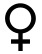	0.40
		girls	2.88	2.48	19					
	B-VIC	boys	2.76	1.75	109					
		girls	3.17	2.16	30					

Notes: 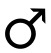 = boys; 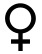 = girls; NI = Non-involved; VIC = Cyber-victim; AGGR = Cyber-aggressor; B-VIC = Cyber-bully/Victim; F.D. = Freedom Degrees; SD = Standard Deviation.

**Table 4:** ANOVA to determine the differences in parenting practices of father according to the role of involvement in cyberbullying.

	Groups	*N*	Mean	SD	F	F.D.	Sig.	*Post Hoc*	Cohen’s *d*
Affection and Communication	NI	1452	4.99	1.06	27.19	(3, 1791)	0.00	NI ≠ VIC	0.61
	VIC	121	4.33	1.37				NI ≠ AGGR	0.59
	AGGR	76	4.36	1.22				NI ≠ B-VIC	0.48
	B-VIC	143	4.47	1.24					
Behavioural Control	NI	1422	4.52	1.22	9.26	(3, 1753)	0.00	NI ≠ AGGR	0.44
	VIC	121	4.32	1.36				NI ≠ B-VIC	0.35
	AGGR	75	3.98	1.28					
	B-VIC	136	4.09	1.31					
Psychological Control	NI	1402	2.90	1.21	10.83	(3, 1727)	0.00	NI ≠ VIC	0.36
	VIC	118	3.34	1.30				NI ≠ B-VIC	0.39
	AGGR	71	3.14	1.21					
	B-VIC	137	3.38	1.19					
Promotion of Autonomy	NI	1415	4.77	1.04	11.79	(3, 1743)	0.00	NI ≠ VIC	0.40
	VIC	115	4.35	1.23				NI ≠ B-VIC	0.39
	AGGR	76	4.48	1.19					
	B-VIC	138	4.36	1.14					
Humour	NI	1472	5.00	0.99	21.49	(3, 1810)	0.00	NI ≠ VIC	0.39
	VIC	121	4.61	1.22				NI ≠ AGGR	0.46
	AGGR	74	4.54	1.18				NI ≠ B-VIC	0.58
	B-VIC	144	4.41	1.21					
Disclosure	NI	1370	4.04	1.39	14.89	(3, 1679)	0.00	NI ≠ VIC	0.38
	VIC	110	3.51	1.55				NI ≠ AGGR	0.42
	AGGR	70	3.46	1.33				NI ≠ B-VIC	0.45
	B-VIC	130	3.41	1.37					
Inductive discipline	NI	1364	3.24	1.72	6.00	(3, 1674)	0.00	NI ≠ B-VIC	0.35
	VIC	113	3.49	1.79					
	AGGR	69	3.66	1.79					
	B-VIC	129	3.84	1.76					
Punishment	NI	1317	1.38	1.65	24.04	(3, 1615)	0.00	NI ≠ VIC	0.36
	VIC	110	1.99	2.06				NI ≠ AGGR	0.58
	AGGR	65	2.35	1.86				NI ≠ B-VIC	0.66
	B-VIC	124	2.49	1.95					
Response Cost	NI	1341	1.88	1.72	14.70	(3, 1656)	0.00	NI ≠ VIC	0.30
	VIC	115	2.41	2.10				NI ≠ AGGR	0.38
	AGGR	70	2.54	1.87				NI ≠ B-VIC	0.52
	B-VIC	131	2.78	1.97					
Supervision	NI	1379	1.59	1.74	21.44	(3, 1696)	0.00	NI ≠ AGGR	0.43
	VIC	115	2.03	1.93				NI ≠ B-VIC	0.67
	AGGR	70	2.35	2.06				VIC ≠ B-VIC	0.36
	B-VIC	133	2.78	2.24					

Notes: NI = Non-involved; VIC = Cyber-victim; AGGR = Cyber-aggressor; B-VIC = Cyber-bully/Victim; F.D. = Freedom Degrees; SD = Standard Deviation.

**Table 5:** Two-way ANOVAS about the differences in father parenting practices according to the interaction between the role of involvement in cyberbullying and gender.

Variable	Group	Sex	Mean	SD	*N*	F	F.D.	Sig.	Post Hoc	Cohen’s *d*
Psychological control	NI	boys	3.04	1.23	661	6.24	(3, 1721)	0.000	NI 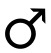 ≠ NI 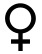	−0.23
		girls	2.76	1.15	736				NI 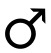 ≠ B-VIC 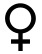	0.58
	VIC	boys	3.42	1.25	60				NI 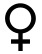 ≠ VIC 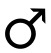	0.57
		girls	3.28	1.35	57				NI 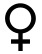 ≠ AGGR 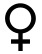	0.15
	AGGR	boys	2.93	1.15	53				NI 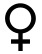 ≠ BVIC 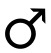	0.44
		girls	3.75	1.20	18				NI 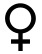 ≠ BVIC 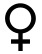	0.86
	B-VIC	boys	3.27	1.14	108					
		girls	3.76	1.31	29					
Promotion of autonomy	NI	boys	4.69	1.04	661	3.26	(3, 1737)	0.021	NI 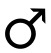 ≠ VIC 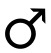	−0.53
		girls	4.83	1.04	749				NI 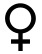 ≠ VIC 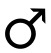	−0.67
	VIC	boys	4.12	1.27	56				NI 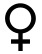 ≠ BVIC 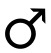	−0.39
		girls	4.56	1.15	58				NI 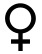 ≠ BVIC 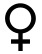	−0.64
	AGGR	boys	4.58	1.20	57					
		girls	4.15	1.12	19					
	B-VIC	boys	4.42	1.00	107					
		girls	4.14	1.53	31					

Notes: NI = Non-involved; VIC = Cyber-victim; AGGR = Cyber-aggressor; B-VIC = Cyber-bully/Victim; F.D. = Freedom Degrees; SD = Standard Deviation.

**Table 6:** Variables included in the regression equation for the different roles of cyber-bullying.

Dependent Variable	Predictors Variables	*B*	Odds Ratio	Sig.
Role of Cyber-victim	Mother’s affection and communication (GS)	−0.25	0.77	0.027
	Father’s affection and communication (GS)	−0.27	0.76	0.010
	Mother’s affection and communication (B)	−0.47	0.62	0.000
	Father’s punishment (G)	0.30	1.35	0.000
Role of Cyber-aggressor	Father’s inductive discipline (GS)	0.24	1.27	0.003
	Father’s affection and communication (GS)	−0.42	0.65	0.000
	Father’s promotion of autonomy (B)	0.59	1.80	0.015
	Father’s affection and communication (B)	−0.71	0.49	0.000
	Mother’s inductive discipline (G)	0.68	1.97	0.000
	Father’s affection and communication (G)	−0.54	0.58	0.004
Role of Cyber-bully/victim	Mother’s punishment (GS)	0.16	1.17	0.029
	Father’s supervision (GS)	0.13	1.14	0.043
	Disclosure to mother (GS)	−0.24	0.78	0.004
	Father’s supervision (B)	0.20	1.23	0.000
	Disclosure to father (B)	−0.26	0.76	0.004
	Mother’s punishment (G)	0.47	1.6	0.000
Role of non-involved	Father’s affection and communication (GS)	0.39	1.48	0.000
	Father’s inductive discipline (GS)	−0.12	0.88	0.016
	Mother’s punishment (GS)	−0.18	0.82	0.000
	Mother’s affection and communication (B)	0.45	1.57	0.000
	Mother’s supervision (B)	−0.13	0.87	0.006
	Father’s affection and communication (G)	0.32	1.84	0.002
	Mother’s punishment (G)	−0.16	0.84	0.074
	Mother’s response cost (G)	−0.24	0.78	0.004

Notes: GS = General sample; B = Boys; G = Girls; *B* = non-standardized coefficient.

## References

[1] Bernete F. (2010). Usos de las TIC, relaciones sociales y cambios en la socialización de las y los jóvenes. Rev. Estud. Juv..

[2] Machimbarrena J.M., Calvete E., Fernández-González L., Álvarez-Bardón A., Álvarez-Fernández L., González-Cabrera J. (2018). Internet Risks: An Overview of Victimization in Cyberbullying, Cyber Dating Abuse, Sexting, Online Grooming and Problematic Internet Use. Int. J. Environ. Res. Public. Health.

[3] Zych I., Ortega-Ruiz R., Del Rey R. (2015). Scientific research on bullying and cyberbullying: Where have we been and where are we going. Aggress. Violent Behav..

[4] Smith P.K., Mahdavi J., Carvalho M., Fisher S., Russell S., Tippett N. (2008). Cyberbullying: Its nature and impact in secondary school pupils. J. Child. Psychol. Psychiatry.

[5] Olweus D., Smith P.K., Morita Y., Jurgen-Tas J., Olweus D., Catalano R., Slee P. (1999). The Nature of School Bullying: A Cross National Perspective.

[6] Ortega R., Mora-Merchán J. (2008). Las redes de iguales y el fenómeno del acoso escolar: Explorando el esquema dominio-sumisión. Infancia Aprendiz..

[7] Ybarra M.L., Espelage D.L., Mitchell K.J. (2014). Differentiating Youth Who Are Bullied From Other Victims of Peer-Aggression: The Importance of Differential Power and Repetition. J. Adolesc. Health.

[8] Dooley J.J., Pyzalski J., Cross D. (2009). Cyberbullying Versus Face-to-Face Bullying: A Theoretical and Conceptual Review. Z. Psychol. J. Psychol..

[9] Patchin J.W., Hinduja S. (2011). Traditional and nontraditional bullying among youth: A test of general strain theory. Youth Soc..

[10] Gini G., Espelage D.L. (2014). Peer Victimization, Cyberbullying, and Suicide Risk in Children and Adolescents. JAMA Pedriatrics.

[11] Twyman K., Saylor C., Taylor L.A., Comeaux C. (2010). Comparing children and adolescents engaged in cyberbullying to matched peers. CyberPsychol. Behav. Soc. Netw..

[12] Del Rey R., Casas J.A., Ortega-Ruiz R. (2016). The impacts of the CONRED Program on different cyberbulling roles. Aggress. Behav..

[13] Gage N.A., Prykanowski D.A., Larson A. (2014). School Climate and Bullying Victimization: A Latent Class Growth Model Analysis. Sch. Psychol. Q..

[14] Navarro R., Serna C., Martínez V., Ruiz-Oliva R. (2013). The role of Internet use and parental mediation on cyberbullying victimization among Spanish children from rural public schools. Eur. J. Psychol. Educ..

[15] Floros G.D., Siomos K.E., Fisoun V., Dafouli E., Geroukalis D. (2013). Adolescent Online cyberbullying in Greece: The Impact of Parental Online Security Practices, Bonding, and Online Impulsiveness. J. Sch. Health.

[16] Lee S.J., Chae Y.G. (2007). Children’s Internet use in a family context: Influence on family relationships and parental mediation. Cyberpsychol. Behav..

[17] Lwin M.O., Stanaland A., Miyazaki A. (2008). Protecting children’s privacy online: How parental mediation strategies affect website safeguard effectiveness. J. Retail..

[18] Chang F.C., Chiu C.H., Miao N.F., Chen P.H., Lee C.M., Chiang J.T., Pan Y.C. (2015). The relationship between parental mediation and Internet addiction among adolescents, and the association with cyberbullying and depression. Compr. Psychiatry.

[19] Baumrind D. (1968). Authoritarian Vs. Authoritative parental control. Adolescence.

[20] Maccoby E.E., Martin J.A., Mussen P.H., Hetherington E.M. (1983). Socialization in the Context of the Family: Parent-Child Interaction. Handbook of Child Psychology: Vol. 4. Socialization, Personality and Social Development.

[21] Martínez I., Murgui S., Garcia O.F., Garcia F. (2019). Parenting in the digital era: Protective and risk parenting styles for traditional bullying and cyberbullying victimization. Comput. Hum. Behav..

[22] Dehue F., Bolman C., Vollink T., Pouwelse M. (2012). Cyberbullying and traditional bullying in relation with adolescents’ perception of parenting. J. CyberTher. Rehabil..

[23] Low S., Espelage D. (2013). Differentiating cyber bullying perpetration from non-physical bullying: Commonalities across race, individual, and family predictors. Psychol. Violence.

[24] Ybarra M.L., Mitchell K.J. (2004). Youth engaging in online harassment: Associations with caregiver-child relationships, Internet use, and personal characteristics. J. Adolesc..

[25] Oliva A., Parra A., Sanchez-Queija I., López F. (2007). Estilos educativos materno y paterno: Evaluación y relación con el ajuste adolescente. An. Psicol..

[26] Manzi C., Regalia C., Pelucchi S., Fincham F.D. (2012). Documenting different domains of promotion of autonomy in families. J. Adolesc..

[27] Kerr M., Stattin H. (2000). What Parents know, How They Know it, and Several Forms of Adolescent Adjustment: Further Support for a Reinterpretation of Monitoring. Dev. Psychol..

[28] Kerr M., Stattin H., Özdemir M. (2012). Perceived Parenting Style and Adolescent Adjustment: Revisiting Directions of Effects and the Role of Parental Knowledge. Dev. Psychol..

[29] Kerr M., Stattin H., Trost K. (1999). To know you is to trust you: Parents’ trust is rooted in child disclosure of information. J. Adolesc..

[30] Fanti K.A., Demetriou A.G., Hawa V.V. (2012). A longitudinal study of cyberbullying: Examining risk and protective factors. Eur. J. Dev. Psychol..

[31] Kokkinos C.M. (2013). Bullying and Victimization in Early Adolescence: Associations with Attachment Style and Perceived Parenting. J. Sch. Violence.

[32] Makri-Botsari E., Karagianni G. (2014). Cyberbullying in Greek adolescents: The role of parents. Procedia Soc. Behav. Sci..

[33] Wang J., Iannotti R.J., Nansel T.R. (2009). School Bullying Among Adolescents in the United States: Physical, Verbal, Relational, and Cyber. J. Adolesc. Health.

[34] Shapka J.D., Law D.M. (2013). Does One Size Fit All? Ethnic Differences in Parenting Behaviors and Motivations for Adolescent Engagement in Cyberbullying. J. Youth Adolesc..

[35] Álvarez-García D., Pérez J.C.N., González A.D., Pérez C.R. (2015). Risk factors associated with cybervictimization in adolescence. Int. J. Clin. Health Psychol..

[36] Law D.M., Shapka J.D., Olson B.F. (2010). To control or not to control? Parenting behaviours and adolescent online aggression. Comput. Hum. Behav..

[37] Dehue F., Bolman C., Völlink T. (2008). Cyberbullying: Youngsters’ Experiences and Parental Perception. Cyberpsychol. Behav..

[38] Fousiani K., Dimitropoulou P., Michaelides M.P., Van Petegem S. (2016). Perceived Parenting and Adolescent Cyber-Bullying: Examining the Intervening Role of Autonomy and Relatedness Need Satisfaction, Empathic Concern and Recognition of Humanness. J. Child. Fam. Stud..

[39] Gómez-Ortiz O., Del Rey R., Casas J.A., Ortega-Ruiz R. (2014). Parenting styles and bullying involvement. Cult. Educ..

[40] Gómez-Ortiz O., Romera E.M., Ortega-Ruiz R. (2016). Parenting styles and bullying. The mediating role of parental psychological aggression and physical punishment. Child. Abuse Negl..

[41] Gómez-Ortiz O., Rey R.D., Romera E.-M., Ortega-Ruiz R. (2015). Los estilos educativos paternos y maternos en la adolescencia y su relación con la resiliencia, el apego y la implicación en acoso escolar. An. Psicol..

[42] Samper-García P., Mestre-Escrivá V., Malonda E., Mesurado B. (2015). Victimización en la escuela: Relación de la crianza y variables funcionales-disfuncionales del desarrollo. Ann. Psicol..

[43] Gryczkowski M.R., Jordan S.S., Mercer S.H. (2010). Differential Relations between Mothers’ and Fathers’ Parenting Practices and Child Externalizing Behavior. J. Child. Fam. Stud..

[44] Cea D’Ancona M.A. (2004). Análisis Multivariable: Teoría y Práctica en la Investigación Social.

[45] Cea D´Ancona M.A. (1996). La Selección de las Unidades de Observación: El Diseño de la Muestra. Metodología Cuantitativa: Estrategias y téCnicas de Investigación Social.

[46] Straus M.A., Fauchier A. (2007). Manual for the Dimensions of Discipline Inventory (DDI).

[47] Calvete E., Gámez-Guadix M., Orue I. (2010). El Inventario de Dimensiones de Disciplina (DDI), Versión niños y adolescentes: Estudio de las prácticas de disciplina parental desde una perspectiva de género. Ann. Psicol..

[48] Del Rey R., Casas J.A., Ortega-Ruiz R., Schultze-Krumbholz A., Scheithauer H., Smith P.K., Thompson F., Barkoukis V., Tsorbatzoudis V., Brighi A. (2015). Structural validation and cross-cultural robustness of the European Cyberbullying Intervention Project Questionnaire. Comput. Hum. Behav..

[49] Bryant F.B., Satorrra A. (2012). Principles and practice of scaled difference Chi-Square testing. Struct. Equ. Model. Multidiscip. J..

[50] Hu L., Bentler P. (1999). Cutoff criteria for fit indexes in covariance structure analysis: Conventional criteria versus new alternatives. Struct. Equ. Model..

[51] Cohen J. (1992). A power primer. Psychol. Bull..

[52] Van Leuween K.G., Fauchier A., Straus M.A. (2012). Assessing Dimensions of Parental Discipline. J. Psychopathol. Behav. Assess..

[53] Inguglia C., Ingoglia S., Liga F., Lo Coco A., Lo Cricchio M.G. (2015). Autonomy and relatedness in adolescence and emerging adulthood: Relationships with parental support and psychological distress. J. Adult Dev..

[54] Nelsen J. (2007). Cómo Educar con Firmeza y Cariño (Disciplina Positiva).

[55] Tisak J., Tisak M.S., Baker E.R., Amrhein K.E., Jensen C. (2017). The Association Among Parental Bonding, Depression, Social Aggression, and Criminal Assault: Are There Gender Differences Between Male and Female Youth Offenders?. J. Interpers. Violence.

[56] Xing X., Wang M., Zhang Q., He X., Zhang W. (2011). Gender Differences in the Reciprocal Relationships Between Parental Physical Aggression and Children’s Externalizing Problem Behavior in China. J. Fam. Psychol..

[57] Perry N.B., Leerkes E.M., Dunbar A.S., Cavanaugh A.M. (2017). Gender and Ethnic Differences in Young Adults’ Emotional Reactions to Parental Punitive and Minimizing Emotion Socialization Practices. Emerg. Adulthood.

[58] Rodrigo M.J. (2016). Quality of implementation in evidence-based positive parenting programs in Spain: Introduction to the special issue. Psychosoc. Interv..

[59] García F., Gracia E. (2009). Is always authoritative the optimum parenting style? Evidence from Spanish families. Adolescence.

[60] Pinquart M. (2017). Associations of parenting dimensions and styles with externalizing problems of children and adolescents: An updated meta-analysis. Dev. Psychol..

[61] Dwairy M., Achoui M., Abouserie R., Farah A. (2006). Adolescent-family conectedness among Arabs: A second cross-regional research study. J. Cross-Cult. Psychol..

